# Feasibility of ANFIS-PSO and ANFIS-GA Models in Predicting Thermophysical Properties of Al_2_O_3_-MWCNT/Oil Hybrid Nanofluid

**DOI:** 10.3390/ma12213628

**Published:** 2019-11-04

**Authors:** Ibrahim M. Alarifi, Hoang M. Nguyen, Ali Naderi Bakhtiyari, Amin Asadi

**Affiliations:** 1 Department of Mechanical and Industrial Engineering, College of Engineering, Majmaah University, Al-Majmaah 11952, Saudi Arabia; i.alarifi@mu.edu.sa; 2 Division of Computational Physics, Institute for Computational Science, Ton Duc Thang University, Ho Chi Minh City 700000, Vietnam; nguyenminhhoang1@tdtu.edu.vn; 3 Faculty of Electrical and Electronics Engineering, Ton Duc Thang University, Ho Chi Minh City 700000, Vietnam; 4 Center for Advanced Laser Manufacturing, School of Mechanical Engineering, Shandong University of Technology, Shandong 255049, China

**Keywords:** thermophysical properties, ANFIS, PSO, GA, MWCNT-Al_2_O_3_ nanoparticles, dynamic viscosity, thermal conductivity, heat transfer performance

## Abstract

The main purpose of the present paper is to improve the performance of the adaptive neuro-fuzzy inference system (ANFIS) in predicting the thermophysical properties of Al_2_O_3_-MWCNT/thermal oil hybrid nanofluid through mixing using metaheuristic optimization techniques. A literature survey showed that the use of an artificial neural network (ANN) is the most widely used method, although there are other methods that showed better performance. Moreover, it was found in the literature that artificial intelligence methods have been widely used for predicting the thermal conductivity of nanofluids. Thus, in the present study, genetic algorithms (GAs) and particle swarm optimization (PSO) have been utilized to search and determine the antecedent and consequent parameters of the ANFIS model. Solid concentration and temperature were considered as input variables, and thermal conductivity, dynamic viscosity, heat transfer performance, and pumping power in both the internal laminar and turbulent flow regimes were the outputs. In order to evaluate and compare the performance of the models, two statistical indices of root mean square error (RMSE) and determination coefficient (R) were utilized. Based on the results, both of the models are able to predict the thermophysical properties appropriately. However, the ANFIS-PSO model had a better performance than the ANFIS-GA model. Finally, the studied thermophysical properties were developed by the trained ANFIS-PSO model.

## 1. Introduction

According to Choi and Eastman [1], the introduction of nanofluids, which are a suspension of nano-sized particles in conventional fluids (i.e., water, ethylene glycol (EG), oil, and so forth), has opened new doors to improve heat transfer rate. After this pioneering study, many researchers conducted different projects on preparation methods [2,3,4], characterization [5,6], thermophysical properties [7,8,9,10,11], heat transfer performance [12,13,14,15,16], and the possible applications [17,18,19] of different nanofluids. Due to the importance of nanofluids, many researchers have reviewed the published literature on different aspects of nanofluids, such as their thermophysical properties [20,21], methods regarding their modeling and simulation [22], and their applications [23,24]. 

No one would disagree that conducting an experimental study on the thermophysical properties and heat transfer of nanofluids is time-consuming and costly. Thus, it would be useful to have a tool to predict the thermophysical properties and heat transfer of nanofluids. Here, artificial intelligence could be utilized. Over the last decade, a growing body of literature has been published on the application of artificial intelligence in predicting the thermophysical properties of different nanofluids [25,26,27]. In this regard, Li et al. [28] employed an adaptive neuro-fuzzy inference system (ANFIS) and an artificial neural network (ANN) to predict the thermophysical properties of SWCNT/Silver-water nanofluid. They proposed a new correlation to predict the thermophysical properties. In another study performed by Hojjat [29], the thermal and hydrodynamic performance of a nanofluid was predicted by ANN method. They used four parameters of thermal conductivity, the Reynolds number, the solid concentration of nanoparticles, and the Prandtl number as the input variables to predict the Nusselt number and pressure drop. They found that there was a good agreement between the experimental data and the ANN output. The mixed convection of a water-based nanofluid with CNT nanoparticles has been modeled using ANFIS by Selimefendigil and Oztop [30]. Alrashed et al. [31] experimentally studied the thermophysical properties of Cu-water nanofluid and employed the ANN method to predict the thermophysical properties. They stated that the ANN has a good capacity in predicting the thermophysical properties of the studied nanofluid. Baghban et al. [32] used different machine learning methods (ANFIS, ANN, and least square support vector machine (LSSVM)) to predict the thermophysical properties of a CNT-water nanofluid. They reported that the LSSVM possesses the best performance over the other studied methods. Adio et al. [33] employed a genetic algorithm-polynomial neural network (GA-PNN) and a fuzzy C-means clustering-based adaptive neuro-fuzzy inference system (FCM-ANFIS) to predict the dynamic viscosity of MgO-EG nanofluid at different temperatures and solid concentrations. They reported that both of the employed methods possessed a good capability to predict the dynamic viscosity of the nanofluid. Table 1 presents a summary of the published literature on the application of artificial intelligence in predicting the thermophysical properties of nanofluids. Moreover, Bahiraei et al. [34] and Ramezanizadeh et al. [35] have reviewed different machine learning methods employed by researchers to predict the thermophysical properties of various nanofluids. 

A literature survey showed that most of the conducted studies evaluated the thermal conductivity of nanofluids by applying the ANN method. Even though many researchers have appropriately used a variety of artificial intelligent methods for predicting the thermophysical properties, the novel artificial intelligent methods (e.g., hybrids and ensembles) have still not been used to fully explore the thermophysical properties. In the present study, the performance of an ANFIS model in predicting the thermophysical properties, heat transfer performance, and pumping power of MWCNT-Al_2_O_3_/oil hybrid nanofluid has been improved through combining it with metaheuristic optimization techniques; genetic algorithms (GAs) and particle swarm optimization (PSO) have been utilized to search and determine the antecedent and consequent parameters of the ANFIS model. The temperature and solid concentration were considered as the input variables, and thermophysical properties (thermal conductivity and dynamic viscosity), heat transfer, and pumping power were the output variables. The performance of the ANFIS models (ANFIS-GA and ANFIS-PSO) were evaluated using two statistical indices of root mean square error (RMSE) and determination coefficient (R).

## 2. Experimental Data

In the present study, the experimental data of thermophysical properties as well as theoretical data of heat transfer performance and pumping power of a hybrid oil-based nanofluid containing MWCNT and Al_2_O_3_ nanoparticle presented by Asadi et al. [46] have been used. They prepared the nanofluid samples by employing the two-step method, which is a method widely used in the literature. The measurement of the thermal conductivity and dynamic viscosity of the nanofluid were done at different temperatures (25–50 °C) and solid concentrations (0.125–1.5 vol.%). Measuring the dynamic viscosity of the nanofluid, they employed a Brookfield cone and plate viscometer, which had been calibrated before starting the experiments. Moreover, they used the KD2 Pro thermal analyzer (Decagon device, Pullman, WA, USA) to measure the thermal conductivity of the nanofluids.

## 3. Adaptive Neuro-Fuzzy Inference System 

Jang was the first researcher who introduced the adaptive neuro-fuzzy inference system (ANFIS) in 1993 [47]. In general, the chief incentive of using ANFIS is to make a powerful mixture of an artificial neural network (ANN) and a fuzzy inference system (FIS) [36]. The FIS is constructed based on the if-then rules, so that the relationship between input and output variables can be determined through the regulations [48]. Hence, it can be fitted as a prediction model for situations when input and output data are highly uncertain; as under these conditions, the uncertainties in the data cannot be considered in classical prediction methods [49]. Principally, two inference systems of Mamdani and Takagi–Sugeno are implemented in fuzzy logic [50]. ANFIS is usually applied based on the inference system of Takagi–Sugeno [51]. 

**Figure 1:**
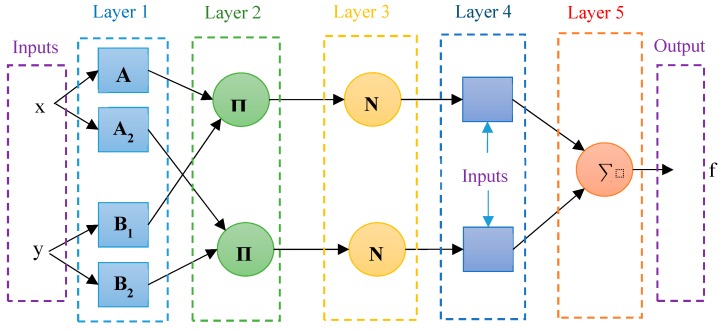
ANFIS Structure.

The ANFIS structure consists of five layers, as displayed in Figure 1. In each layer, the nodes are divided into two forms of adaptable and fixed. In this system, the nodes of layers 2, 3, and 5 (circular nodes) signify fixed nodes, and the nodes of layers 1 and 4 (square nodes), known as adaptive nodes, represent nodes in which parameters are capable to learn [52]. 

In order to explain the rules of each layer, we take two fuzzy if-then rules into consideration as follows:

(1) $$\mathrm{Rule}1:\;\mathrm{if}\;x\;\mathrm{is}\;A1_{\;}\mathrm{and}\;y\;\mathrm{is}\;{B_{1}}_{\;}\mathrm{then}\;f=p_{1}x+q_{1}y+r_{1},$$

(2) $$\mathrm{Rule}2:\;\mathrm{if}\;x\;\mathrm{is}\;A2_{\;}\mathrm{and}\;y\;\mathrm{is}\;B2_{\;}\mathrm{then}\;f=p_{2}x+q_{2}y+r_{2},$$

where *x* and *y* are input variables, A_i_ and B_i_ are fuzzy sets, and *f* is the output (linguistic variables). {*p_i_*, *q_i_*, *r_i_*} are consequent parameters, which should be measured during the ANFIS training process. The function of each layer can be measured as follows: 

Layer 1: In this layer, each node, *i*, is defined by a membership function. The variables in fuzzy logic become fuzzy by means of membership functions. In fact, these membership functions are curves that define how a point in the input space is mapped to a membership value in the interval of [0,1] [53]. Membership functions have various forms; the most common one is the Triangular, Trapzoidum, and Gaussian membership function.

(3) $$O_{1.i}=\sigma_{A_{i}\left(x\right)},$$

(4) $$O_{1.i}=\sigma_{B_{i}\left(x\right)},$$

where *x* is defined as the input of node *i* and *O*_1.i_ is the membership function of *A_i_*, which is usually defined by the Gaussian function as follows:

(5) $$\sigma_{A_{i}\left(x\right)}=exp\left(\frac{-{\left(x-c\right)}^{2}}{\sigma^{2}}\right).$$

In this formula, σ stands for standard deviation and *C* is the center of the Gaussian membership function, which are called antecedent parameters. These parameters are relevant to membership functions, and their value is measured by the optimization algorithm. 

Layer 2: The firing strength of a rule is defined by the following relation:

(6) $$\omega_{i}=\sigma_{A_{i}\left(x\right)}\times \sigma_{B_{i}\left(x\right)}\;i=1.2.$$

Layer 3: The firing strength of each rule is normalized by dividing the firing strength of the *i*th rule to the total firing strength of all rules. 

(7) $$O_{3.i}={\bar{\omega }}_{i}=\frac{\omega_{i}}{\omega_{1}+\omega_{2}}\;i=1.2.$$

Layer 4: The result section of the fuzzy rule is measured as follows:

(8) $$O_{4.i}={\bar{\omega }}_{i}f_{i}={\bar{\omega }}_{i}\left(p_{i}x+q_{i}y+r_{i}\right)\;i=1.2,$$

where {*p_i_*, *q_i_*, r_i_} are the set of consequent parameters, which are computed by the optimization algorithm. 

Layer 5: In this layer, all the outputs of Layer 4 are added to each other.

(9) $$O_{5.i}=\sum_{i=1}^{R}{\bar{\omega }}_{i}f_{i}\;i=1.2.$$

### 3.1. ANFIS Training 

**Figure 2:**
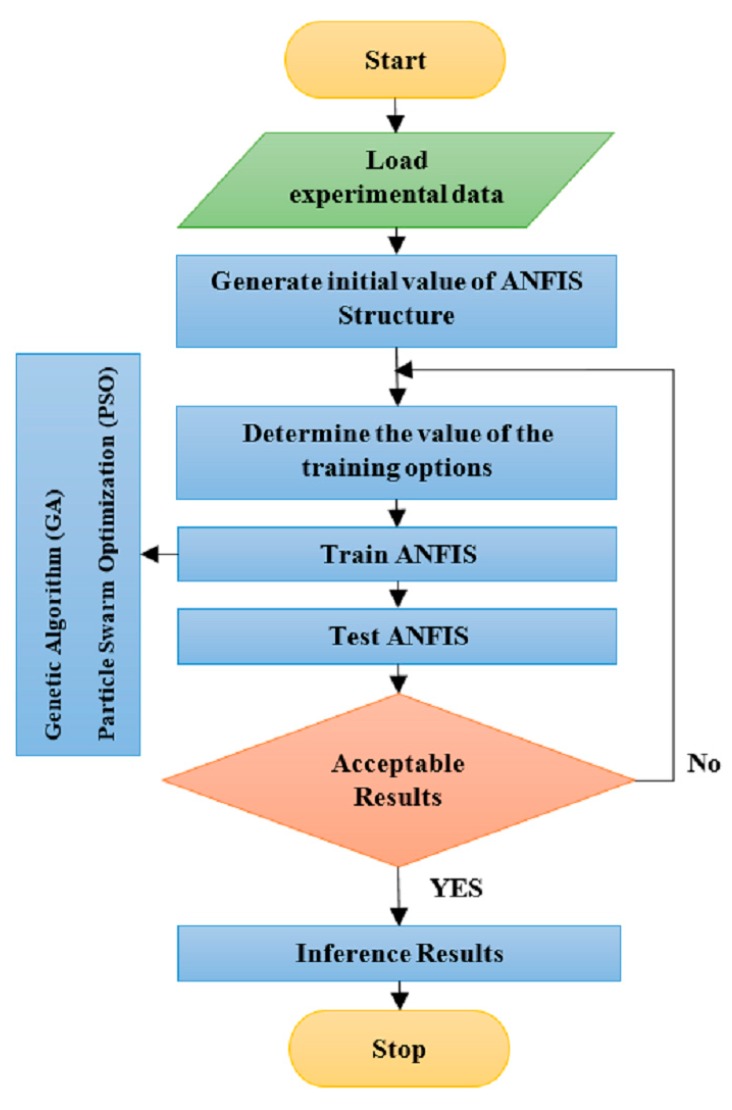
The process of ANFIS training.

In general, two structural parameters of the ANFIS model include antecedent and consequent parameters [54]. The gradient-based methods are usually used to adjust the antecedent and consequent parameters in the ANFIS model [55]. One of the issues with the gradient-based methods is that the answer is placed in local optimality, and convergence rate is slow [56,57]. Metaheuristic optimization algorithms, such as particle swarm optimization (PSO) or the genetic algorithm (GA), can be utilized as an effective solution for the issues relating to the gradient-based methods [58,59,60]. The process of training an ANFIS model using metaheuristic optimization techniques (PSO and GA) is displayed in Figure 2. 

### 3.2. Genetic Algorithm

One of the most effective metaheuristic methods used to find the minimum and maximum points of a target function is the genetic algorithm [61]. This algorithm was first presented by Holland in 1967 and then completed by Goldberg in 1989 [62,63]. Genetic algorithms utilize Darwin’s natural selection principles to find the optimal formula for predicting or matching patterns. Genetic algorithms are frequently great options for random prediction techniques. 

In genetic algorithm solutions, the problem is searched for randomly, step by step. The objective of the search is to find better answers at every stage rather than just the previous one. One of the highlighted features of the genetic algorithm is its ability to run in parallel, which helps it solve complex problems [64]. In this method, the parameters of the search space are first shaped in the form of strings called chromosomes. Each chromosome denotes an answer to the problem in question. Together, the chromosomes form a set called the population, and at the beginning of the operation, the initial population elements are typically selected randomly. The algorithm applies two crossover intersection and mutation functions on population elements iteratively and makes a new population from another one. The answers of a population are usually called the generation. In the end, the favorable answers are produced in the last generation, after the finite repetition. Without a shadow of a doubt, all answers are not necessarily optimal. In order to determine the optimality of each answer, a criterion is used that is called “the objective function.” The target function allocates a value to each population chromosome of one generation, which specifies the suitability of this answer rather than the other answers of the same generation.

### 3.3. Particle Swarm Optimization (PSO)

The PSO algorithm is one of the optimization methods, inspired by nature, which was first invented in 1995 by Hub and Kennedy [65]. This algorithm is mostly utilized to solve numerical optimization issues with very big search space without knowing about the target function gradient [66]. To solve a problem, a population of candidate solutions moves randomly, using a simple formula, into the problem domain. It then explores, aiming to find the optimal global solution (each candidate solution is called a particle).

As in the PSO algorithm, a population of solutions is randomly produced by the algorithm, which look for the answer by moving within the problem domain, in a similar way to the genetic algorithm [67]. Nevertheless, unlike genetic algorithms, in the PSO algorithm, a random velocity is assigned to each potential answer of the optimization problem, or rather each particle, such that in each iteration, any particle is moved regarding its velocity. Furthermore, in the PSO algorithm, unlike the genetic algorithm, the best solution for the optimization problem (from the start of the program to the last repetition) should be stored by each particle. The PSO algorithm is fundamentally appropriate for solving continuous unconstrained maximization problems, like the genetic algorithm [68]. Yet, they can also be utilized to solve optimization problems (including minimization or maximization) in a continuous state with some changes in definition of the function definition [58].

## 4. Results and Discussion

The values of thermal conductivity, as well as dynamic viscosity, heat transfer performance, and pumping power in both the internal laminar and turbulent flow regimes were measured at temperatures of 25, 30, 35, 40, 45, and 50 °C, and volume fractions of 0.125%, 0.25%, 0.5%, 1%, and 1.5% so as to train and test the presented models in this paper. Among all the experimental data, 80% were randomly utilized as training data, and the surplus were used to test the models. 

The two statistical indices correlation coefficient (R), which compares the linear relationship between the experimental and predicted values, and the root mean square of error (RMSE), which compares the deviation between the predicted and actual values through some positive values, were chosen in this study so as to assess and compare the performance of developed models in prediction.

Table 2 demonstrates the parameters of the genetic and the PSO algorithms in which the stopping factor is the number of iterations. The parameters in Table 2 were chosen based on the authors’ experience and through trial and error. It is noteworthy to mention that the RMSE is considered as a target function for the optimization algorithms. 

Table 3 displays the values of the statistical indices computed for any models in predicting thermophysical properties. Inspecting the lowest value of RMSE, it can be concluded that the ANFIS-PSO model shows the best performance in estimating studied thermophysical properties, rather than the ANFIS-GA.

**Figure 3:**
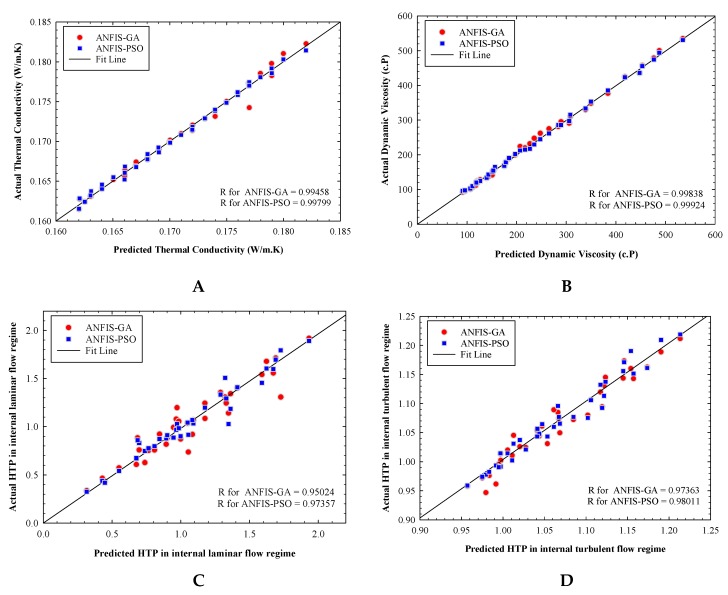

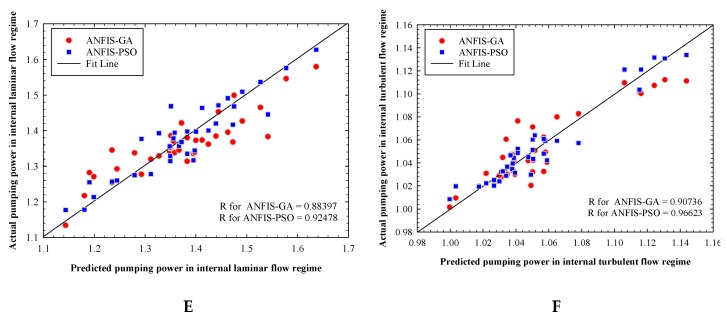
Regression plot for (**A**) thermal conductivity, (**B**) dynamic viscosity, (**C**) internal laminar flow, (**D**) internal turbulent flow, (**E**) internal laminar pumping power, and (**F**) internal turbulent pumping power prediction of Mg(OH)_2_-MWCNT-oil hybrid nanofluid.

In order to perform further examinations, the regression diagram of the experimentally measured values versus the predicted values are displayed in Figure 3. As can be observed, the points are scattered around the fit line (the fit line represents the experimental data), indicating a great adjustment between the model output values and the actual values. It is easy to perceive that having a correlation coefficient close to one, the ANFIS-PSO model gives a minor error compared to the other model in predicting thermophysical properties. 

**Figure 4:**
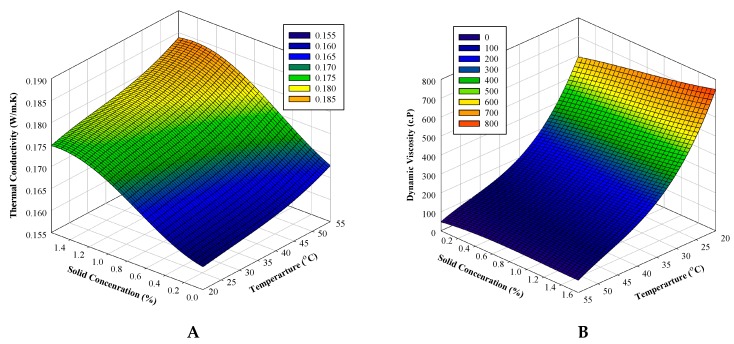

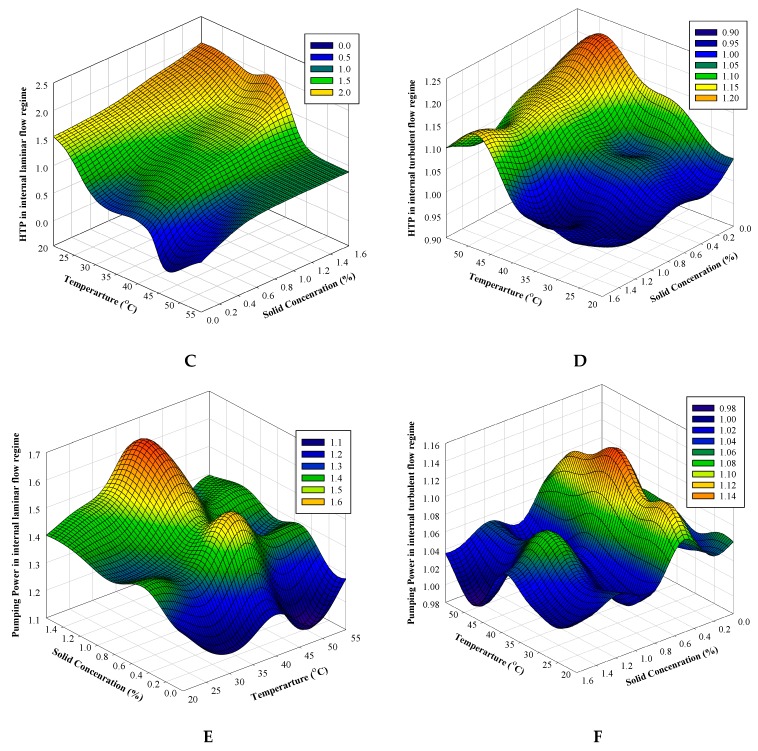
Three-dimensional mesh plot of the developed; (**A**) thermal conductivity, (**B**) dynamic viscosity, (**C**) HTP in the internal laminar flow regime, (**D**) HTP in the internal turbulent flow regime, (**E**) pumping power in the internal laminar flow regime, and (**F**) pumping power in the internal turbulent flow regime using ANFIS-PSO via temperature and solid concentration.

Finally, since the ANFIS-PSO model performed better than the ANFIS-GA model in predicting thermophysical properties, in the present study, this model is utilized as the preferred one to develop the thermophysical properties in different temperatures ranging from 20–50 °C and solid concentrations ranging from 0%–1.6%. Employing the well-trained ANFIS-PSO model, the input data set considered all the states of temperature and solid concentration with increments of 1 °C and 0.1 vol.% in the mentioned ranges, respectively. Regarding the capability of the proposed ANFIS-PSO model in accurate estimation of the thermophysical properties of Al_2_O_3_-MWCNT-oil hybrid nanofluid, the outputs of the ANFIS-PSO model regarding temperature and solid concentration are shown in a three-dimensional mesh plot (Figure 4). As can be seen, the ANFIS-PSO model produces a smooth surface that shows the high accuracy of the model. The values of the studied thermophysical properties in the defined temperature and solid concentration data set obtained from the ANFIS-PSO models are displayed in Figure 4. 

## 5. Concluding Remarks

In this paper, in order to improve the performance of the ANFIS model to predict the thermophysical properties of Al_2_O_3_-MWCNT/thermal oil hybrid nanofluid, PSO and genetic algorithms were utilized. In this method, the antecedent and consequent parameters of the ANFIS model were regulated by the searching mechanism of optimal values of genetic algorithms and PSO. In order to train and test the models, data sets of experimental thermophysical properties measured by Asadi et al. [46] at different temperatures and solid concentration were employed. Thermal conductivity coefficient, dynamic viscosity, heat transfer performance, and pumping power in both internal laminar and turbulent flow regimes were utilized as predictive parameters. Based on the outcomes, we can conclude that the use of metaheuristic algorithms can be helpful in improving the ANFIS model training process. The results demonstrate that both models are capable of predicting thermophysical properties, appropriately. However, based on comparisons between models, the ANFIS-PSO model produced better results in comparison to the ANFIS-GA model. Finally, using the ANFIS-PSO model at a temperature range of 10–50 °C and a volume fraction of 0%–2%, the studied thermophysical properties were developed.

## Figures and Tables

**Table 1:** A summary of the recently published literature on using neural networks in predicting the thermophysical properties of nanofluids.

Reference	Nanofluid	Studied Properties	Method
Bagherzadeh et al. [26]	F-MWCNT-Fe_3_O_4_/EG	Thermal conductivity	Enhanced ANN
Alrashed et al. [36]	Diamond- and MWCNT-COOH/water	Viscosity, density, and thermal conductivity	ANFIS and ANN
Bahrami et al. [27]	Fe-CuO/EG-water	Dynamic viscosity	ANN
Safaei et al. [37]	ZnO-TiO_2_/EG	Thermal conductivity	ANN and Curve-fitting
Ghasemi et al. [38]	COOH-MWCNT/EG	Thermal conductivity	ANN and Curve-fitting
Kannaiyan et al. [39]	Al_2_O_3_-SiO2/water	Thermal conductivity and density	ANN
Moradikazerouni et al. [40]	SWNT-EG	Thermal conductivity	ANN and curve-fitting
Hemmat Esfe et al. [41]	Al_2_O_3_/Water-EG (60%–40%)	Thermal conductivity	ANN
Eshgarf et al. [42]	MWCNT-SiO_2_/EG-Water	viscosity	ANN
Vakili et al. [43]	CuO/Water-EG	Thermal conductivity	ANN
Maddah et al. [44]	MWCNT-Carbon (60%–40%)/SAE 10W40-SAE 85W90 (50-50%)	Viscosity	ANN
Vafaei et al. [45]	MgO-MWCNT/EG	Thermal conductivity	ANN

**Table 2:** Genetic algorithm (GA) and particle swarm optimization (PSO) algorithm parameters.

GA Parameters		PSO Parameters	
Population Size	20	Population Size	20
Maximum Number of Iterations	1000	Maximum Number of Iterations	1000
Crossover Percentage	0.7	Inertia Weight	1
Mutation Percentage	0.5	Inertia Weight Damping Ratio	0.99
Mutation Rate	0.1	Personal Learning Coefficient	1
Selection Pressure	8	Global Learning Coefficient	2
Gamma	0.2		

**Table 3:** The values of the root mean square of error (RMSE) computed for the models.

Model	ANFIS-GA		ANFIS-PSO	
Data Set	Train	Test	Train	Test
Thermal Conductivity	3.91 ×10^−4^	1.44 × 10^−3^	3.47 × 10^−4^	5.11 × 10^−4^
Dynamic Viscosity	7.07	8.55	4.56	7.31
HTP in internal laminar flow regime	8.38 × 10^−2^	2.89 × 10^−1^	5.68 × 10^−2^	2.14 × 10^−1^
HTP in internal turbulent flow regime	1.37 × 10^−2^	2.24 × 10^−2^	1.11 × 10^−2^	2.35 × 10^−2^
PP in internal laminar flow regime	5.59 × 10^−2^	5.64 × 10^−2^	3.99 × 10^−2^	6.22 × 10^−2^
PP in internal turbulent flow regime	2.45 × 10^−2^	1.30 × 10^−2^	8.66 ×10^−3^	1.12 × 10^−2^
